# Chalcone-Derived Nrf2 Activator Protects Cognitive Function via Maintaining Neuronal Redox Status

**DOI:** 10.3390/antiox10111811

**Published:** 2021-11-15

**Authors:** Yuting Cui, Yue Xiong, Hua Li, Mengqi Zeng, Yan Wang, Yuan Li, Xuan Zou, Weiqiang Lv, Jing Gao, Ruijun Cao, Lingjie Meng, Jiangang Long, Jiankang Liu, Zhihui Feng

**Affiliations:** 1 Center for Mitochondrial Biology and Medicine, The Key Laboratory of Biomedical Information Engineering of Ministry of Education, School of Life Science and Technology, Xi’an Jiaotong University, Xi’an 710049, China; yutingcui111@stu.xjtu.edu.cn (Y.C.); xw_xjtu@163.com (Y.X.); huali2014@xjtu.edu.cn (H.L.); zmq2010122024@stu.xjtu.edu.cn (M.Z.); wy13572504396@stu.xjtu.edu.cn (Y.W.); gl824705609@stu.xjtu.edu.cn (W.L.); gaojing005@stu.xjtu.edu.cn (J.G.); jglong@mail.xjtu.edu.cn (J.L.); 2 Institute of Basic Medical Science, Xi’an Medical University, Xi’an 710021, China; yuanli@xiyi.edu.cn; 3 National and Local Joint Engineering Research Center of Biodiagnosis and Biotherapy, The Second Affiliated Hospital of Xi’an Jiaotong University, Shannxi 710004, China; zuseon@xjtu.edu.cn; 4 Shaanxi Provincial Clinical Research Center for Hepatic and Splenic Diseases, The Second Affiliated Hospital of Xi’an Jiaotong University, Shannxi 710004, China; 5 MOE Key Laboratory for Nonequilibrium Synthesis and Modulation of Condensed Matter, School of Science, Xi’an Jiaotong University, Xi’an 710049, China; rjcao@mail.xjtu.edu.cn (R.C.); menglingjie@mail.xjtu.edu.cn (L.M.); 6 University of Health and Rehabilitation Sciences, Qingdao 266071, China; 7 Frontier Institute of Science and Technology, Xi’an Jiaotong University, Xi’an 710049, China

**Keywords:** phase II enzymes, Nrf2, Akt, hippocampus, mitochondrial function

## Abstract

NF-E2-related factor 2 (Nrf2), the key transcription regulator of phase II enzymes, has been considered beneficial for neuronal protection. We previously designed a novel chalcone analog, 1-(2,3,4-trimethoxyphenyl)-2-(3,4,5-trimethoxyphenyl)-acrylketone (Tak), that could specifically activate Nrf2 in vitro. Here, we report that Tak confers significant hippocampal neuronal protection both in vitro and in vivo. Treatment with Tak has no significant toxicity on cultured neuronal cells. Instead, Tak increases cellular ATP production by increasing mitochondrial function and decreases the levels of reactive oxygen species by activating Nrf2-mediated phase II enzyme expression. Tak pretreatment prevents glutamate-induced excitotoxic neuronal death accompanied by suppressed mitochondrial respiration, increased superoxide production, and activation of apoptosis. Further investigation indicates that the protective effect of Tak is mediated by the Akt signaling pathway. Meanwhile, Tak administration in mice can sufficiently abrogate scopolamine-induced cognitive impairment via decreasing hippocampal oxidative stress. In addition, consistent benefits are also observed in an energy stress mouse model under a high-fat diet, as the administration of Tak remarkably increases Akt signaling-mediated antioxidative enzyme expression and prevents hippocampal neuronal apoptosis without significant effect on the mouse metabolic status. Overall, our study demonstrates that Tak protects cognitive function by Akt-mediated Nrf2 activation to maintain redox status both vivo and in vitro, suggesting that Tak is a promising pharmacological candidate for the treatment of oxidative neuronal diseases.

## 1. Introduction

Memory and learning impairments are the most typical features in neuronal diseases, resulting from dysfunction and loss of neurons in the brain tissues, particularly the hippocampus [1]. Within the brain, the hippocampus, basal forebrain, and amygdala are extremely vulnerable to oxidative damage due to high oxygen consumption and an abundance of unsaturated fatty acids and metal ions while maintaining a relatively low antioxidant capacity [2,3]. Cumulative oxidative stress can not only result in oxidized molecules but also impair key organelles, such as mitochondria, by reducing the production of ATP required for neuron survival and metabolism [4]. Hippocampal oxidative stress is thereby acknowledged as a crucial causative factor of deterioration in the learning and memory performance.

It is well-documented that learning and memory are closely related to glutamatergic and cholinergic neurotransmission in the mammalian central nervous system (CNS) [5]. Normally, glutamate, as a major endogenous excitatory neurotransmitter, is responsible for glutamatergic neurotransmission [6]. However, an excess of extracellular glutamate can lead to neurotoxicity via ionotropic glutamate receptor-mediated excitotoxicity and cystine/glutamate antiporter-mediated oxidative stress, contributing to the development of many acute and chronic brain diseases [7,8,9,10]. Chronic exposure of neurons to glutamate results in persistent activation of postsynaptic glutamate receptors, destabilizing the intracellular Ca^2+^ balance and eventually causing neuronal death [11]. The mechanism of glutamate-induced oxidative toxicity has been extensively characterized, and extracellular glutamate accumulation has been speculated to inhibit cystine uptake by reversing the action of the cystine/glutamate antiporter, leading to glutathione (GSH) deprivation, which further promotes the accumulation of intracellular reactive oxygen species (ROS) [12]. An increase in ROS activates 12-lipoxygenase and accelerates Ca^2+^ influx [13], promoting mitochondrial dysfunction-associated ATP deprivation, apoptosis-inducing factor (AIF) nuclear translocation, DNA fragmentation, and cell death [14,15,16].

Scopolamine, a nonselective muscarinic acetylcholine receptor antagonist, is clinically suggested as an anti-muscarinic and anti-cholinergic drug. However, it was reported that scopolamine could promote glutamate release and rapidly increase extracellular glutamate levels, subsequently triggering a burst of glutamate transmission [17], resulting in cognitive impairment [18,19], and the scopolamine-induced cognitive impairment presented strong positive correlation with changes in neuronal oxidative status and mitochondrial function [20,21,22,23]. In addition, consumption of a high-fat diet (HFD) has been shown to be a risk factor for glutamate-induced oxidative stress and mitochondrial dysfunction, contributing to metabolic dysfunction-associated neuronal death and cognitive decline [24,25]. Both scopolamine and HFD intervention have been widely used as animal models for the study of neuronal oxidative stress-associated cognitive impairment.

Chalcones, one of the subclasses of the flavonoid family, are precursors of flavones in the biosynthesis of flavonoids and have been reported to exhibit a broad spectrum of pharmacological properties [26]. We previously designed and screened a novel chalcone analog, 1-(2,3,4-trimethoxyphenyl)-2-(3,4,5-trimethoxyphenyl)-acrylketone (Tak, Supplemental Figure S1), which could significantly induce phase II enzyme expression in human retinal pigment epithelia (ARPE-19) cells via promoting nuclear factor-E2-related factor 2 (Nrf2) nuclear translocation due to its unique chemical structure [27]. It is thus tempting to speculate that Tak, as an Nrf2 activator, may exhibit a neuroprotective effect through attenuating oxidative stress and recovering mitochondrial function. The present study was dedicated to investigating the neuronal protection of Tak and the possible underlying mechanisms in vitro and in vivo.

## 2. Materials and Methods

### 2.1. Chemicals and Reagents

Glutamate (G3291), LY294002 (L9908), JC-1 (T4069), ATP bioluminescent cell assay kit (MAK190), and 3-(4,5-dimethyl-2-thiazolyl)-2,5-diphenyl-2-H-tetrazolium bromide (M5655) were obtained from Sigma (St. Louis, MO, USA). Antibodies against p-Akt (9271), Akt (9272), p-Erk1/2 (9106), Erk1/2 (4695), p-p38 (4511), p38 (8690), p-JNK1/2 (9255), JNK1/2 (9252), BDNF (47808), caspase 3 (14220), and β-Actin (3700) were purchased from Cell Signaling Technology (Danvers, MA, USA). Antibodies against Complex I (459130), Complex II (459230), Complex III (459140), Complex IV (459600), and Complex V (459240) were purchased from Thermo Fisher (Rockford, IL, USA). Antibodies against NQO-1 (sc-376023), HO-1 (sc-390991), SOD1 (sc-101523), and SDO2 (sc-133134) were purchased from Santa Cruz (Dallas, TX, USA). Antibodies against NGF (ab52918) and Nrf2 (ab92946) were purchased from Abcam (Cambridge, UK). Cell culture medium (11965092), H2DCF-DA (C369), bicinchoninic acid protein assay kit (23252), Mito-Tracker^®^ Green FM (M7514), and MitoSOX^TM^ Red Mitochondrial Superoxide Indicator (M36008) were purchased from Life Technologies (San Diego, CA, USA). Secondary antibodies including Goat Anti-Rabbit IgG (111-035-003), Goat Anti-Mouse IgG (115-035-003), and Rabbit Anti-Goat IgG (305-035-003) were purchased from Jackson ImmunoResearch (West Grove, PA, USA). A FITC Annexin V Apoptosis Detection Kit (556547) was obtained from BD Biosciences (San Jose, CA, USA). Hoechst staining kit (C1011) was purchased from Beyotime (Shanghai, China). TRIzol Reagent (9108), PrimeScript RT-PCR Kit (6210A), and TB Green Premix Ex Taq II kit (RR820B) were purchased from Takara (Shiga, Japan). Tak was synthesized from 1,2,3-trimethoxybenzene following a previously published study [27], and Tak at the purity of 96.1% was used in the present study. The synthetic scheme and structure of Tak are shown in Figure S1.

### 2.2. Cell Culture

HT22 mouse hippocampal neuronal cell line was purchased from Sigma (SCC129, St. Louis, MO, USA). SH-SY5Y human neuroblastoma cell line was purchased from ATCC (CRL-2266, Manassas, VA, USA). Cells were cultured in DMEM medium (11965092, Life Technology, Rockford, IL, USA) supplemented with 10% fetal calf serum (30-2030, ATCC), 100 U/mL penicillin G sodium (P3032, Sigma), and 100 μg/mL streptomycin sulfate (11860038, Thermo Fisher). The cells were maintained at 37 °C in a humidified atmosphere with 5% CO_2_. The medium was changed every two days. HT22 and SH-SY5Y cells were used within 8 generations.

### 2.3. Assessment of Cell Viability

Cell viability was analyzed using a 3-(4,5-dimethyl-2-thiazolyl)-2,5-diphenyl-2-H-tetrazolium bromide (MTT) assay. Both HT22 and SH-SY5Y cells were seeded at a density of 3.5 × 10^4^ cells/mL in 96-well plates, allowing cells to attach and grow for 24 h, followed by indicated compound treatments. After the treatment, cultured medium was discarded and living cells were cultured with 200 μL of MTT–DMEM solution (0.5 mg/mL in serum free medium) for 4 h. The MTT solution was then discarded, and 200 μL DMSO was added to each well to dissolve the MTT formazan. The absorbance was measured at a wavelength of 590 nm using a microplate fluorometer (Fluoroskan Ascent; Thermo Fisher Scientific, Inc., Rockford, IL, USA).

### 2.4. JC-1 Assay for MMP

Mitochondrial membrane potential (MMP) was analyzed, following a previously published study [27]. Briefly, cells were cultured at a density of 3.5 × 10^4^ cells/mL in 96-well plates. After treatment, MMP was analyzed using the lipophilic cationic probe 5,5′,6,6′-terachloro-1,1′,3,3′-tetraethyl-imidacarbocyanine iodide (JC-1, 5 μg/mL in serum-free medium). The cells were washed with PBS once and scanned with a microplate fluorometer (Fluoroskan Ascent; Thermo Fisher) at a 488-nm excitation wavelength and 538- and 590-nm emission wavelengths to measure green and red JC-1 fluorescence, respectively. The red/green fluorescence intensity ratio reflects the MMP.

### 2.5. Oxygen Consumption Rate Analysis

Oxygen consumption rates of living cells were analyzed with the Seahorse method (Seahorse Bioscience, Santa Clara, CA, USA) [28]. Cells were seeded in XF 24-well microplates. After treatment, oxygen consumption was measured using extracellular flux analysis. The final concentrations of the mitochondrial inhibitors were 1 μM antimycin A, 4 μM carbonyl cyanide-4-(trifluoromethoxy) phenylhydrazone (FCCP) and 4 μM oligomycin. Basal respiration was the baseline oxygen consumption reading before the compounds were injected. Basal, maximal, ATP-linked, and nonmitochondrial respiration and the reserve capacity were adjusted according to the protein concentration in each well, which was measured using the bicinchoninic acid method.

### 2.6. Mitochondrial Superoxide Analysis

Cells were cultured at a density of 5 × 10^4^ cells/mL in 24-well plates. After treatment, the generation of mitochondrial superoxides was observed using a mitochondrial superoxide indicator (MitoSOX^TM^ Red), and the mitochondria were assessed using Mito-Tracker^®^ Green FM, following a previous study [27]. Briefly, the cells were stained with 500 nM Mito-Tracker^®^ Green FM in serum-free medium for 30 min, followed by incubation with 10 μM MitoSOX^TM^ Red in serum-free medium for 10 min. After washing with PBS, the cells were observed by laser scanning confocal microscopy (Zeiss, Jena, Germany).

### 2.7. ATP Analysis

The ATP content was determined using an ATP bioluminescent cell assay kit according to the manufacturer’s instructions. Cells were lysed with 0.5% Triton X-100 in 100 mM glycine buffer (pH 7.4). The supernatant was collected after centrifugation at 21,000× *g* for 10 min at 4 °C. Then, 40 μL of the supernatant was transferred to an appropriate bioluminescence plate. The mixture was agitated for 5 min before the addition of 160 μL reaction solution. The luminescence was measured and normalized to the protein content.

### 2.8. Hoechst Staining

Hoechst staining was employed to identify apoptotic cells by condensed and fragmented nuclei [29]. Briefly, cells were plated onto coverslips at a density of 9 × 10^4^ cells/mL in 6-well plates. After treatment, the cells were fixed with 4% formaldehyde for 30 min at room temperature, followed by washes with PBS. The cells were incubated with the membrane-permeable fluorescent dye Hoechst for 30 min and then observed using fluorescence microscopy (Olympus, Tokyo, Japan) with a peak excitation wavelength of 340 nm.

### 2.9. Annexin V-FITC/PI Double Staining Assay

Cells in the early and late apoptotic stages were analyzed by an Annexin V-FITC/PI double staining assay. Early apoptotic cells showed the externalization of phosphatidylserine (PS) on the outer leaflets of the plasma membrane. Annexin V had a high affinity for PS. Cells were plated at a density of 9 × 10^4^ cells/mL in 6-well plates. When reaching confluence of 80–90%, cells were exposed to Tak or glutamate treatment for the indicated time, then both floating and attached cells were all collected and washed twice with PBS. Cells were resuspended in 500 μL binding buffer, followed by staining with 10 μL Annexin V and 5 μL propidium iodide (PI) in the dark at room temperature for 15 min. The stained cells were analyzed immediately using a FACS flow cytometry analyzer (Beckman Coulter, Indianapolis, IN, USA) with emission filters at wavelengths of 488–530 nm for green fluorescence of Annexin V (FL1) and 488–630 nm for red fluorescence of PI (FL2). A total of at least 10,000 cells per sample were acquired to ensure adequate data. The lower left quadrant indicates the normal cells (Annexin V−, PI−), the lower right quadrant indicates early apoptotic cells (Annexin V+, PI−), and the upper right quadrant indicates late apoptotic cells (Annexin V+, PI+).

### 2.10. Cell Cycle Analysis

Cells were plated at a density of 9 × 10^4^ cells/mL in 6-well plates. When cells were grown to 80–90% confluence, cells were exposed to Tak treatment for 24 h. At the end of the treatment, the cells were serum starved for 3 h. After synchronization, the floating and attached cells were collected and centrifugated at 4 °C 1000× *g* for 5 min, and then cell pellets were washed twice with PBS and resuspended in 500 μL PBS with the addition of 4.5 mL 70% ethanol, followed by incubation at −20 °C for at least 2 h. Afterwards, the cells were collected and centrifugated at 1000× *g* for 5 min. Cell pellets were resuspended in 500 μL 1 mg/mL PI solution (0.1% Triton X-100, 100 μg/mL Rnase A, and 40 μg/mL PI) for 30 min on ice. A total of at least 10,000 cells per sample were recorded and monitored by a FACS flow cytometry analyzer (Beckman Coulter, Brea, CA, USA).

### 2.11. Measurements of Superoxide Dismutase (SOD) Activity, Catalase (CAT) Activity, Glutathione (GSH) Level and Malondialdehyde (MDA) Level

SOD activity, CAT activity, GSH level, and MDA level were measured using commercially available kits (Jiancheng, Nanjing, China) according to the manufacturer’s instructions. The cell or hippocampus tissue samples were dissected rapidly and homogenized in ice-cold 0.9% saline (pH 7.4). After centrifugation, supernatants were collected for the determination of SOD activity, CAT activity, GSH level, and MDA level. SOD and CAT activities were expressed as units (U) per milligram of protein. GSH and MDA levels were calculated by means of a calibration curve and normalized to the protein concentration.

### 2.12. Western Blot Analysis

Samples were lysed with western and immunoprecipitation (IP) lysis buffer (P10013, Beyotime, China). The lysates were homogenized, and the homogenates were centrifuged at 13,000× *g* for 12 min at 4 °C. The supernatants were collected, and the protein concentrations were determined with a bicinchoninic acid (BCA) protein assay kit. Equal aliquots (20 μg) of the protein were separated by 10% SDS-PAGE, transferred to pure nitrocellulose membranes, and blocked with 5% nonfat milk in TBST buffer (8 g/L NaCl, 2.42 g/L Tris, 0.1% Tween20, PH 7.6). The membranes were incubated with primary antibodies at 4 °C overnight. Then, the membranes were incubated with secondary antibodies at room temperature for 1 h. Chemiluminescent detection was performed using an ECL western blotting detection kit (Thermo Fisher, Rockford, IL, USA). The results were analyzed by Quantity One software (Bio-Rad, Shanghai, China) to obtain the optical density ratio of the target proteins relative to β-actin. The antibodies used in the present study were: p-Akt (1:1000), Akt (1:1000), p-Erk1/2 (1:1000), Erk1/2 (1:1000), p-p38 (1:1000), p38 (1:1000), p-JNK1/2 (1:1000), JNK1/2 (1:1000), β-Actin (1:10000), Complex I (1:1000), Complex II (1:1000), Complex III (1:1000), Complex IV (1:1000), Complex V (1:1000), NQO-1 (1:700), HO-1 (1:700), SOD1 (1:700), SOD2 (1:700), BDNF (1:1000), NGF (1:1000), caspase 3 (1:1000), Goat Anti-Rabbit IgG (1:3000), Goat Anti-Mouse IgG (1:3000), and Rabbit Anti-Goat IgG (1:3000).

### 2.13. Real-Time Quantitative PCR

Total RNA was isolated using TRIzol Reagent followed by treatment with chloroform and precipitation with 2-propanol. The total RNA pellet was then washed with 75% ethanol and resuspended in water. The RNA was subjected to reverse transcription using a PrimeScript RT-PCR Kit, and quantitative real-time PCR analysis (CFX96, Bio-Rad, Hercules, CA, USA) of the genes of interest was conducted using a TB Green Premix Ex Taq II kit. Relative gene expression was calculated using the 2^−ΔΔCt^ method. The data were normalized to the expression level of β-actin in percentage, and control in each group was normalized to 100%. Primers are shown in Supplementary Materials (Accession gene IDs: 15368, 18104, 14629, 14630, 12359, 20655, 20656, 11461) (Table S1).

### 2.14. Animal Experiments

For the scopolamine-induced dementia mouse model, 12-week-old male C57BL/6J mice weighing 25–30 g were purchased from Vital River Laboratory Animal Technology Co., Ltd. (Beijing, China). After acclimatization for 1 week, the mice were randomly assigned to following four groups (*n* = 8 in each group): (1) mice were administered with 0.9% saline (Control), (2) mice were administered with the vehicle followed by intraperitoneal (i.p.) injection of 1 mg/kg scopolamine (Sco), (3) mice were intraperitoneally administered with 15 mg/kg Tak prior to intraperitoneal injection of 1 mg/kg scopolamine (Sco + Tak 15 mg/kg), and (4) mice were intraperitoneally administered with 50 mg/kg Tak prior to intraperitoneal injection of 1 mg/kg scopolamine (Sco + Tak 50 mg/kg). The doses of 15 and 50 mg/kg were determined following a previous study showing the protective effect of another chalcone derivative in mice at the dose of 30 mg/kg [19]. Tak was dissolved in 0.4 % DMSO in 0.9% saline and intraperitoneally administered once daily for 9 consecutive days. Hereafter, scopolamine was dissolved in 0.9% saline and intraperitoneally injected to induce cognitive deficit 30 min before each behavioral task.

For the HFD-induced diabetic mouse model, 4-week-old male C57BL/6J mice were purchased from the SLAC Laboratory Animal Co., Ltd. (Shanghai, China). After 1 week of acclimatization, the mice were randomly divided into four groups (*n* = 8 in each group) as follows: (1) mice fed a normal diet (control, 10% kcal fat content, D12492, Research Diets, New Brunswick, NJ, USA), (2) mice fed a high-fat diet (HFD, 60% kcal fat content, D12450, Research Diets), (3) mice fed a HFD with 10 mg/kg/day Tak (taken the long-term feeding into consideration, we changed the dose of Tak from 15 mg/kg/day to 10 mg/kg/day), and (4) mice fed a HFD with 50 mg/kg/day Tak. The HFD feeding proceeded for a total of 8 weeks, and intraperitoneal injections of the vehicle and Tak (0.4% DMSO in PBS) were administered from week 5 to week 8. All animals were housed in a temperature (25–28 °C)- and humidity (60%)-controlled animal room and maintained on a 12-h light/12-h dark cycle with food and water provided during the experiments. Animal procedures were approved by the Xi’an Jiaotong University Animal Care and Use Committee (XJTU-2018-033 for HFD model, XJTU-2019-021 for scopolamine model). All the methods were performed in accordance with approved guidelines, and all efforts were made to minimize the suffering and the number of animals used in this study.

### 2.15. Open Field Test

The open field test is the behavioral test to evaluate locomotion, based on the natural tendency of rodents to explore new environments and to avoid open and brightly lit spaces [30]. The open field chamber is an open area, and a video camera is positioned at the top of chamber. The mice were habituated to the testing room for at least 30 min prior to testing. In the trial, each mouse was placed into the chamber center and allowed to explore freely for 3 min. In the next 15 min, the total distance and average speed were automatically recorded by an animal tracking program (AnyMaze, Shanghai, China). After each trial, the chamber was cleaned and dried in order to avoid any olfactory cues.

### 2.16. Y Maze Test

The Y maze test is based on the natural tendency of rodents to prefer entering a new arm rather than returning to an arm that has just been explored [31]. The Y maze apparatus consists of three identical arms placed at 120° around an equilateral triangular platform in the center, and a video camera is fixed at the top of Y maze apparatus. The mice were habituated to the testing room for at least 30 min prior to testing. In the trial, the mouse was placed at one arm end, facing the short wall, and allowed to move freely through the maze during a 5 min session. After each trial, the Y maze apparatus was cleaned and dried. The sequence and number of arm entries were recorded by AnyMaze (Shanghai, China). An arm entry was completed when the hind paws of the mouse were completely placed in the arm. A triplet was defined as the mouse’s respective alternations between the arms, including spontaneous alternation (every arm was mentioned in a triplet), alternate arm return (the first and third arms were the same), or same arm return (the first and second arms were the same). A spontaneous alternation was defined as consecutive entries into each arm of the Y maze without any repeats. The percentage of spontaneous alternations was calculated based on the following formula: spontaneous alternation percentage = number of spontaneous alternation/number of triplets. The number of arm entries per trial is used as an indicator of locomotor activity.

### 2.17. Novel Object Recognition Test

This test is used to assess short-term spatial recognition memory, based on the natural tendency of rodents to spend more time exploring an unfamiliar stimulus than a familiar one [31]. The general procedure consists of three sessions: the habituation, memory acquisition, and memory recall phases. In habituation phase (2 days), the mice were allowed to freely explore the arena without objects. In the training trial of the memory acquisition phase, the mouse was allowed to explore the arena with two identical objects placed on opposite sides of the cage for 10 min. The cage and objects were cleaned at the end of each trial. During a retention interval of 3 h, the mice were kept individually in a cage until the retention trial. In the retention trial of the memory recall phase, one of the previously familiar objects was substituted for a novel, unfamiliar one. The mouse was allowed to explore the area with two differential objects placed in opposite sides of cage for 10 min. Exploration was defined as sniffing or touching the object with the nose and/or forepaws at a distance of no more than 2 cm. Sitting and standing or leaning on the objects were not considered as an exploration. A discrimination index in the memory recall phase was calculated as time spent exploring the “novel” object compared with the “familiar” object relative to the total time spent exploring the two objects, according to the formula:

discrimination index = (ETNO − ETFO)/(ETNO + ETFO)

ETNO = exploration time of “novel” object; ETFO = exploration time of “familiar” object.

### 2.18. Morris Water Maze (MWM) Test

MWM test is used to assess hippocampal-dependent spatial learning and reference memory [32]. The MWM is a white circular pool with a diameter of 122 cm and a height of 51 cm, located in a room with ample surrounding visual cues. The pool is filled with water containing white dye to a depth of 30 cm (24 ± 1 °C). The pool is conceptually divided into four quadrants. A white platform is centered in one of the quadrants and submerged 1 cm below the water surface so that it is invisible at water level. One day before the acquisition trial, the mice were given a pre-training trial for 60 s in the absence of the platform to acclimatize to the situation. Thereafter, in the acquisition trial, each mouse was subjected to four trials each day for four subsequent days. In each acquisition trial, the mouse was gently placed into the water facing the edge of the pool in a randomly selected quadrant which was changed in each trial per day. If the mouse reached the platform within 60 s, it was permitted to stay on the platform for 5 s to remember the position of the goal in relation to surrounding cues. If the mouse did not find the platform within 60 s, it was gently guided to the platform and allowed to stay there for 15 s. To assess memory consolidation, a probe trial was conducted 24 h after the last acquisition trial. The platform was removed from the pool. The mouse was placed into the pool side opposite to the target quadrant and allowed to freely swim for 60 s. A video camera connected to corresponding software monitored the behavior of the mice in the pool, including the escape latency to the platform, cumulative path length, average speed in each acquisition trial, crossing numbers over the target quadrant, and the time spent and distance traveled in the target quadrant.

### 2.19. Mitochondrial Complex Activity Determination

Hippocampus tissues were collected, resuspended in 1 mL of hypotonic lysis buffer (10 mmol/L NaCl, 2.5 mmol/L MgCl_2_, and 10 mmol/L Tris base (pH 7.5)), and homogenized on ice with a glass homogenizer (Fisher Scientific, Pittsburgh, PA, USA). The homogenates were then centrifugated at 1300× *g* for 5 min at 4 °C. The supernatant was centrifugated at 17,000× *g* for 15 min at 4 °C, and the mitochondrial pellet was resuspended in 100 μL of isotonic buffer (210 mmol/L mannitol, 70 mmol/L sucrose, 5 mmol/L Tris base, and 1 mmol/L EDTA-Na_2_ (pH 7.5)). Assays for reduced nicotinamide adenine dinucleotide (NADH)–ubiquinone reductase (complex I), ubiquinol–cytochrome c reductase (complex III), and Mg^2+^-ATPase (complex V) activities were performed using the activity kits according to our previous method [28].

### 2.20. Statistical Analysis

All cell experiments were repeated at least three times. The data are presented as the mean ± S.E.M. All statistical analyses were performed using SPSS 23 software. The significance of differences was assessed by unpaired Student’s t-test or one-way ANOVA followed by Tukey’s multiple comparisons test. For all comparisons, the level of significance was set at *p* < 0.05.

## 3. Results

### 3.1. Validation of Tak Cytotoxicity on Cultured Neuronal Cells

**Figure 1:**
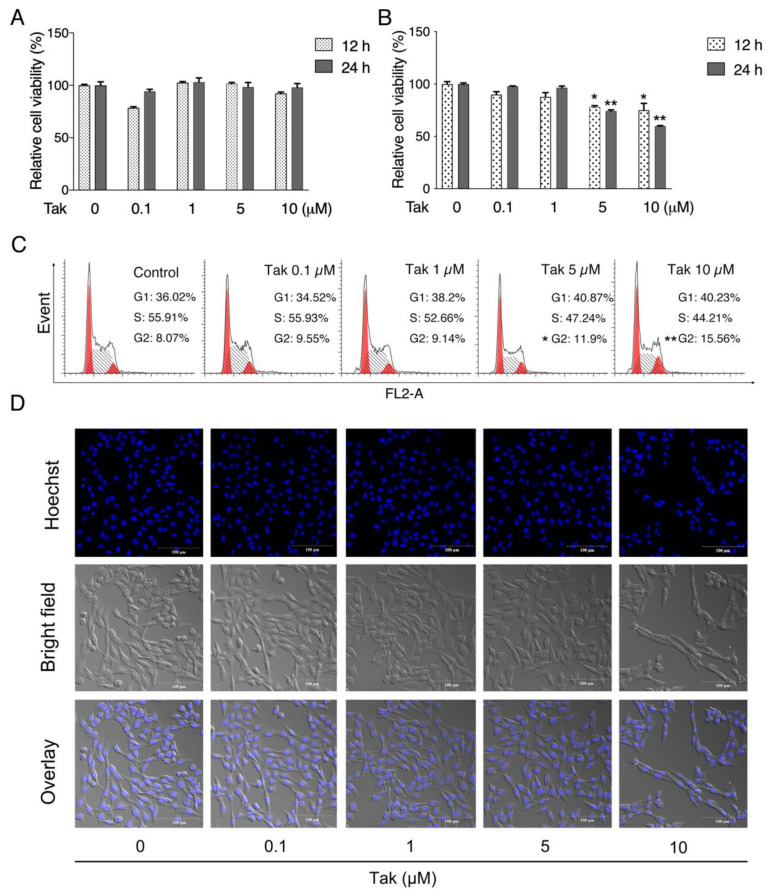
The effect of Tak on cell viability. (**A**) HT22 cells were treated with Tak at concentrations of 0.1, 1, 5, and 10 μM for 12 and 24 h, and cell viability was analyzed. (**B**) SH-SY5Y cells were treated with Tak at concentrations of 0, 0.1, 1, 5, and 10 μM for 12 and 24 h, and cell viability was analyzed. (**C**) Flow cytometry analysis of the cell cycle in SH-SY5Y cells treated with Tak for 12 h. (**D**) Hoechst staining of SH-SY5Y cells treated with Tak for 12 h. The values are presented as the mean ± S.E.M. from at least three independent experiments. * *p* < 0.05 and ** *p* < 0.01 vs. the control.

Since Tak is a new synthetic compound, the biological effects of Tak have not been validated in neuronal cells. Therefore, we analyzed the effects of Tak on cultured HT22 murine hippocampal cells and SH-SY5Y human neuroblastoma cells. Tak had no significant effects on the viability of HT22 cells, but it slightly and significantly decreased viability of SH-SY5Y cells at both 5 and 10 μM after 12 and 24 h treatment (Figure 1A,B). Further cell cycle analysis indicated that Tak induced G2 phase arrest in SH-SY5Y cells (Figure 1C) without significant changes in the cell nucleus and morphology (Figure 1D), suggesting that cell cycle arrest primarily contributes to slightly lowered SH-SY5Y cell viability after Tak treatment. These data suggest that Tak has no significant toxic effects on HT22 and SH-SY5Y cells.

### 3.2. Tak Augments Mitochondrial Activities

**Figure 2:**
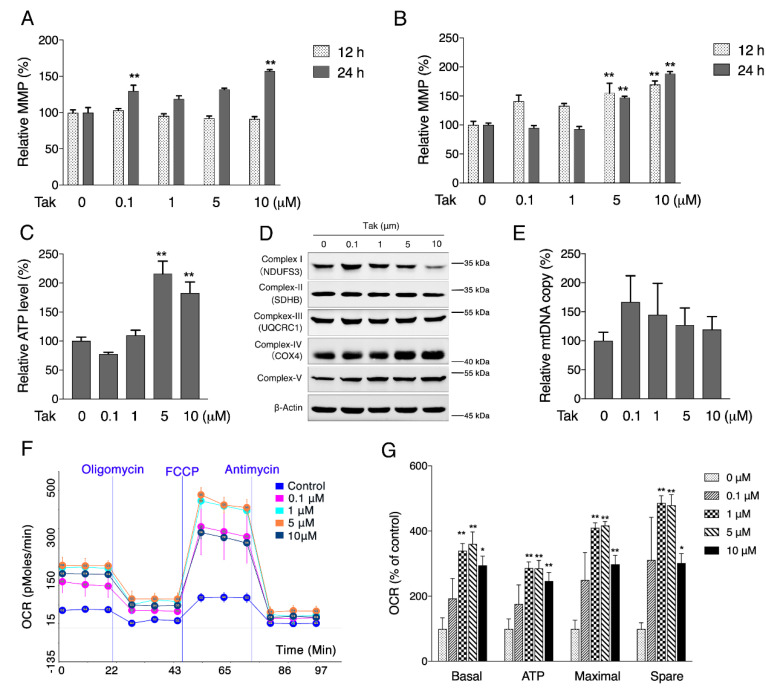
The effect of Tak on mitochondrial activities. (**A**) SH-SY5Y cells were treated with Tak at concentrations of 0, 0.1, 1, 5, and 10 μM for 12 and 24 h, and the MMP was assessed by JC-1 staining. (**B**) HT22 cells were treated with Tak at concentrations of 0, 0.1, 1, 5, and 10 μM for 12 and 24 h, and the MMP was assessed by JC-1 staining. HT22 cells were treated with Tak for 24 h, and the cellular ATP level (**C**), mitochondrial complex subunit expression (**D**), mtDNA copy number (**E**), and mitochondrial oxygen consumption rate ((**F**): experimental program; (**G**): statistical analysis) were analyzed. The values are presented as the mean ± S.E.M. from at least three independent experiments. * *p* < 0.05 and ** *p* < 0.01 vs. the control.

Although the brain only accounts for approximately 2% of the total body weight, it utilizes approximately 20% of total body oxygen intake, which requires a highly dynamic and functional mitochondrial network [33]. MMP is crucial in maintaining the normal function of mitochondria. Tak significantly increased the MMP of SH-SY5Y cells after 24 h of treatment (Figure 2A), while in HT22 cells, Tak could significantly increase MMP after both 12 h and 24 h treatment at the doses of 5 and 10 μM, suggesting an overall beneficial potential of Tak for mitochondria (Figure 2B). Further analysis showed that Tak dose-dependently increased cellular ATP content (Figure 2C). Interestingly, the mitochondrial DNA copy number and complex subunit expression were not affected by Tak (Figure 2D,E). Seahorse analysis of mitochondrial oxygen consumption showed that Tak enhanced mitochondrial respiration capacity, including basal, maximal, ATP potential, and spare respiration (Figure 2F,G), after 24 h of treatment, indicating that enhanced oxygen consumption contributed to improved ATP production by Tak.

### 3.3. Tak Improves Redox Status by Activating Phase II Enzymes

**Figure 3:**
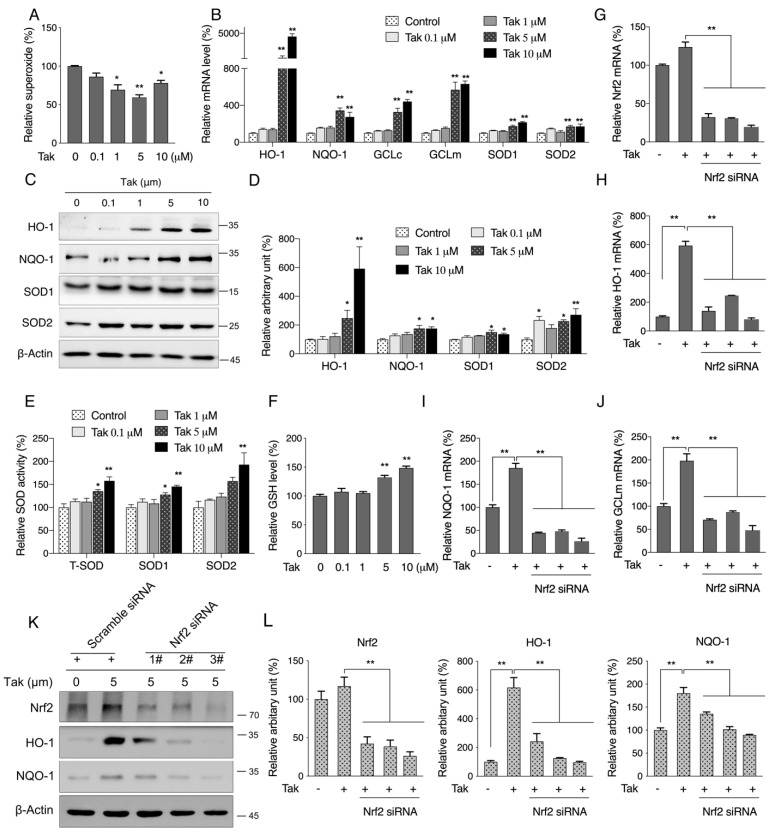
Tak improves redox status by activating phase II enzymes. (**A**) HT22 cells were treated with Tak at concentrations of 0, 0.1, 1, 5, and 10 μM for 24 h, and mitochondrial superoxide was analyzed. HT22 cells were treated with Tak for 6 h for analysis of phase II enzymes mRNA expression (**B**), and 24 h for analysis of protein expression ((**C**): western blot images; (**D**): statistical analysis), SOD activities (**E**), and GSH level (**F**). HT22 cells were treated with three pairs of specific Nrf2 siRNA for 48 h followed by 5 μM Tak treatment for 6 h; the mRNA expression levels of Nrf2 (**G**), HO-1 (**H**), NQO-1 (**I**), and GCLm (**J**) were analyzed by qRT-PCR. HT22 cells were treated with three pairs of specific Nrf2 siRNA for 48 h followed by 5 μM Tak treatment for 24 h; the protein levels of Nrf2, HO-1, and NQO-1 were analyzed by western blot ((**K**): western blot images; (**L**): statistical analysis). The values are presented as the mean ± S.E.M. from at least three independent experiments. * *p* < 0.05 and ** *p* < 0.01 vs. the control or between the connected groups.

To explore the mechanism that contributes to improved mitochondrial activity, we first analyzed the mitochondrial redox status, which is one of the major factors that affect mitochondrial function. Analysis of mitochondrial superoxide showed that Tak dose-dependently decreased ROS levels (Figure 3A). Given the primary contribution of phase II enzymes for maintaining cellular redox status, we thereby analyzed expression levels of endogenous phase II enzymes. Data showed that the mRNA levels of heme oxygenease-1 (HO-1), NAD(P)H: quinone oxidoreductase (NQO-1), γ-glutamyl-cysteine ligase catalytic (GCLc) and modifier (GCLm) subunits, catalase, superoxide dismutase 1 (SOD1), and superoxide dismutase 2 (SOD2) were consistently induced by Tak after 6 h of treatment (Figure 3B). Increased protein expressions (Figure 3C,D), SOD activities (Figure 3E), and cellular GSH levels were observed in response to Tak treatment (Figure 3F). These phase II enzymes have been reported to be regulated by Nrf2, which is known as nuclear factor (erythroid-derived-2)-like 2. To confirm that Tak activates phase II enzymes through Nrf2, three pairs of specific Nrf2 siRNA were transfected into cells prior to Tak treatment. As shown in Figure 3G, the siRNA treatments significantly decreased Nrf2 mRNA expression levels as expected, and Tak-induced HO-1, NQO-1, and GCLm expressions were further abolished by Nrf2 siRNAs (Figure 3H–J); consistent expression pattern was also observed at the protein levels of Nrf2, HO-1, and NQO-1 (Figure 3K,L), indicating that the activation of phase II enzymes by Tak was mediated through Nrf2.

### 3.4. Tak Inhibits Glutamate-Induced Cell Apoptosis

**Figure 4:**
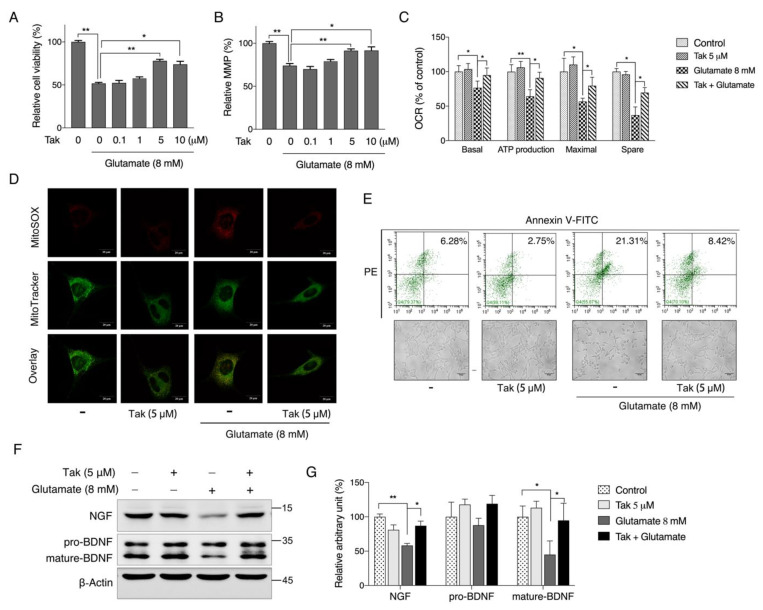
Tak inhibits glutamate-induced cell apoptosis. HT22 cells were treated with Tak at concentrations of 0, 0.1, 1, 5, and 10 μM for 24 h, followed by glutamate treatment (8 mM) for 12 h, and cell viability (**A**), MMP (**B**), and oxygen consumption rate (**C**) was analyzed. HT22 cells were treated with Tak at a concentration of 5 μM for 24 h, followed by glutamate treatment (8 mM) for 12 h, and the mitochondrial superoxide level was assessed by fluorescent confocal microscopy (**D**), cell apoptosis was analyzed by flow cytometry (**E**), and protein expressions of NGF and BDNF were analyzed by western blot ((**F**): western blot images; (**G**): statistical analysis). The values are presented as the mean ± S.E.M. from at least three independent experiments. * *p* < 0.05 and ** *p* < 0.01 between the connected groups.

Glutamate treatment induced time- and dose-dependent loss of HT22 cell viability, and 50% of the cell viability loss was induced by 8 mM treatment for 12 h (Figure S2). Therefore, we used this dose to verify the protective effects of Tak. As shown in Figure 4A, pretreatment with 5 and 10 μM Tak conferred significant protection against glutamate-induced cell viability loss. Consistently, glutamate significantly reduced the MMP, which was markedly improved by 5 and 10 μM Tak (Figure 4B). Seahorse analysis indicated that the mitochondrial respiration capacities, including basal, maximal, ATP potential, and spare respirations, were all decreased by glutamate and partially restored by 5 μM Tak pretreatment (Figure 4C). Furthermore, fluorescence microscopy analysis with MitoSOX showed that the level of mitochondrial superoxide was markedly increased by glutamate, while pretreatment with Tak significantly inhibited the overproduction of mitochondrial superoxide (Figure 4D). Considering that the loss of cell viability and mitochondrial dysfunction are closely associated with cell death, cell apoptosis was thereby analyzed using an Annexin V/PI flow cytometry. As shown in Figure 4E, glutamate markedly augmented the proportion of annexin V and PI double-stained cells to 21%, which was restored to 8% by Tak pretreatment. In addition, neuron-specific proteins including NGF, pro-BDNF, and mature BDNF were markedly repressed by glutamate, whereas they were normalized by Tak pre-treatment (Figure 4F,G). These data suggest that Tak could inhibit glutamate-induced oxidative neurotoxicity via enhancing the antioxidant defense and the mitochondrial function.

### 3.5. Tak Activates Phase II Enzymes through Akt Signaling for Neuronal Protection

**Figure 5:**
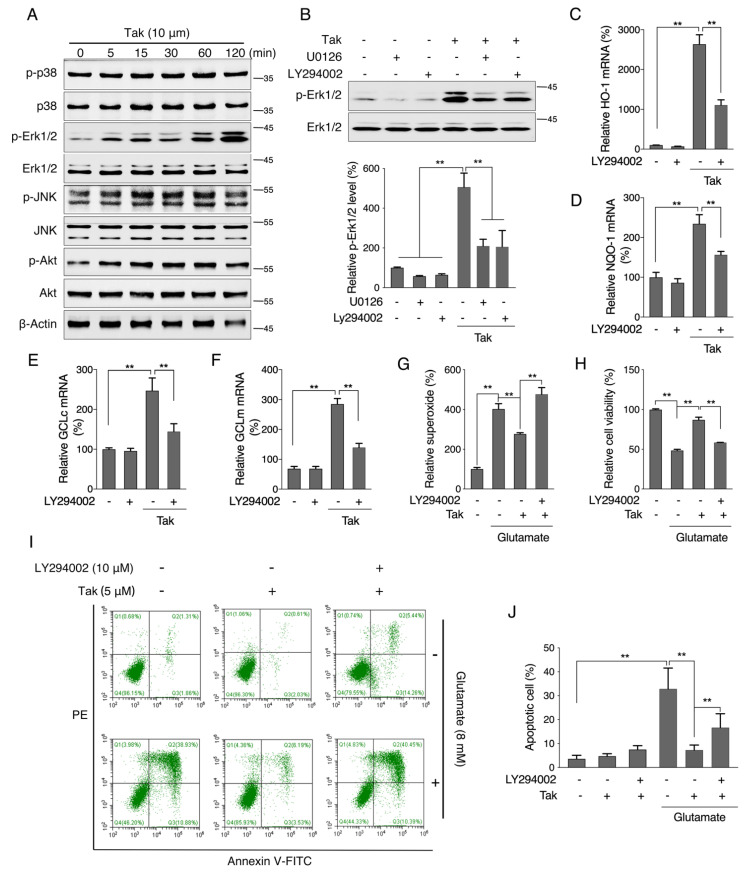
Tak activates phase II enzymes through Akt signaling. (**A**) HT22 cells were treated with 10 μM Tak for 0, 5, 15, 30, 60, and 120 min, and kinase activation was analyzed by western blot. (**B**) HT22 cells were treated with Tak for 30 min with or without LY294002 and U0126, and p-Erk1/2 was analyzed by western blot. HT22 cells were treated with 5 μM Tak for 6 h with or without LY294002, and the mRNA expression levels of HO-1 (**C**), NQO-1 (**D**), GCLc (**E**), and GCLm (**F**) were analyzed by qRT-PCR. HT22 cells were treated with 5 μM Tak for 24 h with or without LY294002, followed by glutamate treatment for 12 h, mitochondrial superoxide (**G**), cell viability (**H**), and cell apoptosis ((**I**): flow cytometry images; (**J**): statistical analysis) were analyzed. The values are presented as the mean ± S.E.M. from at least three independent experiments. * *p* < 0.05 and ** *p* < 0.01 between the connected groups.

To further explore the upstream pathway that regulates phase II enzymes by Tak, we treated HT22 cells in a time-dependent manner and found that Tak effectively increased the levels of p-Akt and p-Erk without affecting other MAP kinases (Figure 5A). Employment of Akt and Erk inhibitors showed that the phosphorylation of Erk was regulated by Akt activation (Figure 5B). Inhibition of Akt activity with LY294002 was found to significantly suppress the expression of phase II enzymes, including HO-1, NQO-1, GCLc, and GCLm (Figure 5C–F), while inhibiting Erk activity failed to block the induction of expression of phase II enzymes (Figure S3A,B). Meanwhile, Tak sufficiently improved glutamate-inhibited Akt phosphorylation, while it had no effect on glutamate-suppressed Erk1/2 phosphorylation (Figure S3C,D), suggesting an Akt signaling-dependent protection of Tak against glutamate toxicity. Further assays confirmed that Akt inhibition completely abolished the protective effect of Tak against the glutamate challenge (Figure 5G–J).

### 3.6. Tak Improves Scopolamine-Induced Cognitive Impairment in Mice

**Figure 6:**
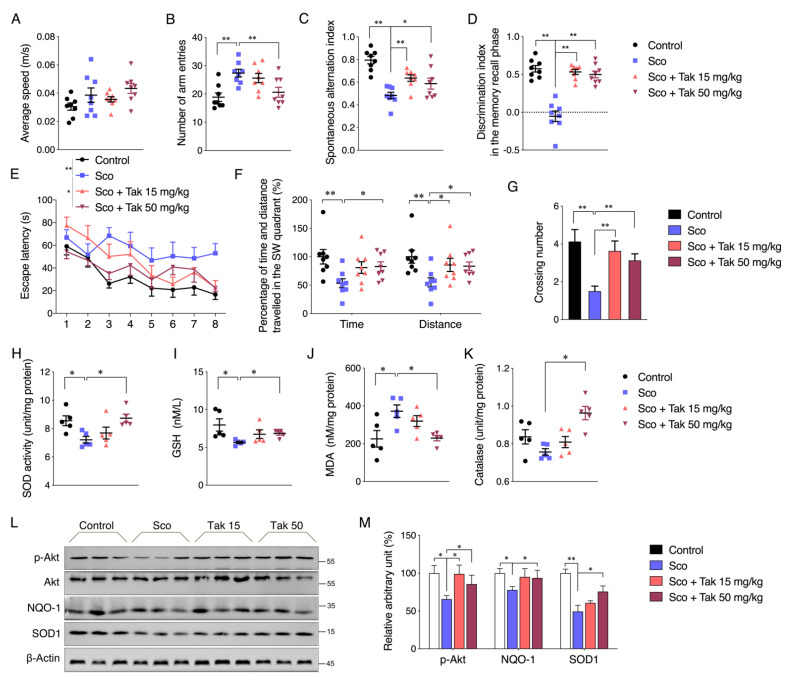
Tak supplementation ameliorates scopolamine-induced learning and memory impairments. Mice were intraperitoneally administered with Tak at the dose of 15 and 50 mg/kg/day for 9 days followed by 1 mg/kg scopolamine injection 30 min before behavioral tests. (**A**) Travelling speed at open field. Total number of arm entries (**B**) and spontaneous alternation index (**C**) in the Y maze. (**D**) Discrimination index in the new object recognition test. (**E**) Escape latency of acquisition trial in the MWM test. (**F**) Percentage of time and distance spent in SW quadrant of probe trial. (**G**) Number of platform crossings in probe trial. The hippocampus tissues were collected after the treatment for analysis of (**H**) SOD activity, (**I**) GSH level, (**J**) MDA level, (**K**) CAT activity, and protein levels of p-Akt, NQO-1, and SOD1 ((**L**): western blot images; (**M**): statistical analysis). The values are presented as the mean ± S.E.M. *n* = 8 for behavioral tests, *n* = 5 for biochemical tests. * *p* < 0.05 and ** *p* < 0.01 between the connected groups. Sco: Scopolamine.

To further investigate the neuronal protective effect of Tak in vivo, scopolamine was administrated to induce mouse cognitive impairment, as established by previous studies [18,19]. The open field test revealed comparable locomotor activity among four groups (Figure 6A). The Y maze test showed increased numbers of arm entries and reduced spontaneous alternation percentage in the scopolamine-treated group, which were all alleviated by the Tak supplement (Figure 6B,C). Meanwhile, the novel object recognition test revealed a dramatic decline in the discrimination index in scopolamine-treated mice, which failed to discriminate the novel object relative to the familiar object, while those with the Tak supplement showed significant improvement, indicating a protection of cognitive function (Figure 6D). In addition, consistent cognitive improvement was also observed in the MWM test, including decreased escape latency over four consecutive days, increased percentage of time and distance traveled in the target quadrant, and increased crossing numbers in the acquisition trial (Figure 6E–G). Further analysis of hippocampus tissues showed decreased SOD activity and GSH levels and increased MDA levels in the scopolamine-challenged mice which were all improved by Tak supplement (Figure 6H–J). Though catalase activity was not altered after administration of the scopolamine challenge, the Tak supplement group still showed a significant increase of catalase activity compared to the scopolamine-treated group, suggesting an overall enhanced antioxidative capacity in mice hippocampi with Tak (Figure 6K). Meanwhile, Akt phosphorylation was decreased by scopolamine treatment and accompanied by decreased phase II enzyme expression, which were all effectively improved by the Tak supplement (Figure 6L,M). Taken together, these data suggest that Tak exerts cognitive improvement and neuronal redox status via maintaining Akt-mediated phase II enzyme expression in scopolamine-challenged mice.

### 3.7. Tak Protects Hippocampal Neurons under Metabolic Stress via Akt/Nrf2 Signaling in Mice

**Figure 7:**
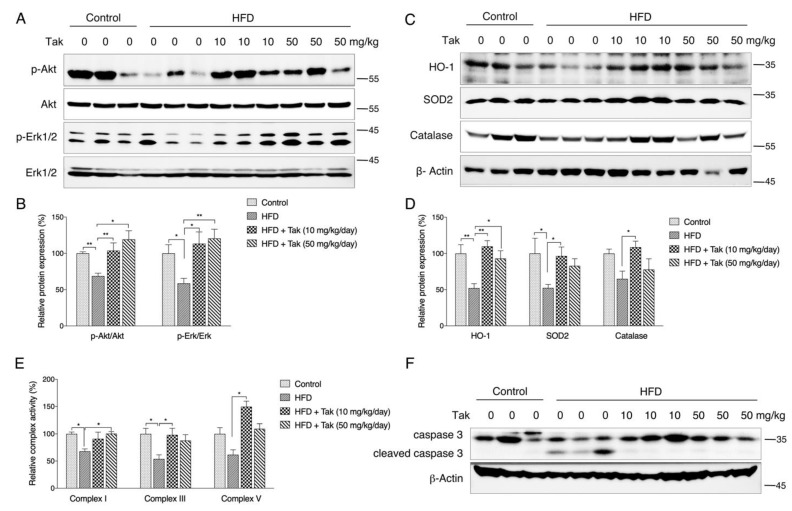
The effects of Tak on hippocampal neurons. Mice were fed a chow diet or HFD with or without Tak supplementation at doses of 10 and 50 mg/kg/day for 8 weeks, and the hippocampus tissues was collected for analysis. p-Akt and p-Erk were analyzed by western blot ((**A**): western blot images; (**B**): statistical analysis). HO-1, SOD2, and catalase were analyzed by western blot ((**C**): western blot images; (**D**): statistical analysis). (**E**) Mitochondrial complex activities. (**F**) Caspase cleavage was analyzed by western blot. The values are presented as the mean ± S.E.M. (*n* = 8). * *p* < 0.05 and ** *p* < 0.01 between the connected groups.

The consumption of HFD has been shown to be a risk factor contributing to metabolic dysfunction-associated neuronal death and cognitive decline. Therefore, in addition to the scopolamine model, the HFD feeding was exercised to evaluate the protective effect of Tak in vivo. As shown in Figure S4, HFD feeding for 8 weeks significantly increased body weight gain compared to the normal diet group, and Tak supplementation at two doses had no significant effects (Figure S4A–C). Consistent with the body weight observations, serum analysis showed that the HFD significantly increased serum FFA, total cholesterol, HDL cholesterol, and LDL cholesterol levels, which were not affected by Tak intervention (Figure S4D–G). These data suggest that HFD-induced metabolic abnormalities are not improved by Tak intervention. Although Tak showed no significant effects on obese mouse metabolism, the Akt and Erk phosphorylation was notably significantly increased by Tak in the hippocampus, which was consistent with the in vitro cellular observations (Figure 7A,B). Further analysis showed that the HFD significantly decreased the expression levels of hippocampal phase II enzymes, such as HO-1, SOD2, and catalase, which were all improved by Tak (Figure 7C,D). Mitochondrial complex activities were analyzed using isolated hippocampal mitochondria, and decreased complex I, III, and V activities were observed in the HFD group, which were all improved by Tak treatment (Figure 7E). Since decreased mitochondrial function is a key factor contributing to apoptosis-associated cell death, we analyzed the caspase 3 level and found that the HFD group significantly promoted the cleavage of caspase 3, which was sufficiently blocked by Tak treatment (Figure 7F). These data suggest that Tak could ameliorate HFD-induced neuronal oxidative stress and apoptosis activation independent of systemic metabolic status.

## 4. Discussion

Synaptic plasticity is the key cellular mechanism involved in learning and memory, which requires glutamatergic neurons to form a neural circuit in the hippocampus. However, excessive excitation of glutamatergic neurons leads to glutamate accumulation in the extracellular compartment, followed by a number of deleterious consequences, including the generation of free radicals and the activation of mitochondrial permeability transition, contributing to neuronal death [34]. In addition, clinical use of the anti-cholinergic drug scopolamine and long-term HFD intake were suggested to disrupt glutamate homeostasis, resulting in increased oxidative stress, mitochondrial dysfunction, and cognitive impairment [18,19,24,25]. Targeting oxidative stress and mitochondrial protection is thereby considered a popular strategy for neuronal survival and cognitive improvement [12,35,36].

The Nrf2 signaling pathway, which orchestrates key cellular antioxidant response mechanisms, has great potential for protecting cells against oxidative stress-mediated brain diseases associated with cognitive impairment [37]. Studies have indicated that overexpression of Nrf2 induced memory improvement in a mouse model of Alzheimer’s disease [38], while impaired Nrf2 signaling and increased cerebral oxidative stress exacerbated deleterious effects of obesity on cognitive performance [24]. Chalcones, biosynthetic precursors of flavonoids, have gained increasing attention for their derivates which could regulate Nrf2 activity [39]. The ability of chalcone derivatives activating Nrf2 was markedly improved with one or two electron-withdrawing groups on phenyl ring B and up to three methoxyl and/or hydroxyl groups on phenyl ring A [40]. We recently synthesized and screened a new chalcone analog that contains three methoxyl groups on both phenyl rings A and B, which effectively activated Nrf2 in vitro [27]. In the current study, both HT22 and SH-SY5Y cell lines were cultured to evaluate the potential toxicity of Tak. Notably, Tak had no significant toxic effect except for slight cell cycle arrest in SH-SY5Y cells. Since the HT22 cell line is an immortalized mouse hippocampal cell line that lacks functional ionotropic glutamate receptors, similar to the undifferentiated neuronal stem cell line, it expresses neuron-specific enolase and neurofilament proteins [41], making it an excellent candidate for a glutamate-induced neuronal oxidative stress model for compound screening.

Considering that mitochondria are the key organelles for neuronal function and survival, we evaluated the effect of Tak on mitochondrial function and found that Tak significantly increased the MMP, cellular ATP level, and oxygen respiration capacity. However, these notable benefits were not attributed to mitochondrial biogenesis. It is estimated that 1–3% of mitochondrial oxygen consumed during the process of oxidative phosphorylation is incompletely reduced, resulting in the formation of superoxide anions, which are a predominant form of ROS produced in mitochondria, and their accumulation could induce oxidative stress to suppress mitochondrial activity [41]. We hypothesized that the improvement in mitochondrial function by Tak was due to its enhanced redox status, which was confirmed by measuring mitochondrial ROS. Phase II enzymes are known as the endogenous antioxidative system that is regulated by Nrf2, which forms heterodimers with small oncogene family proteins for the selective recognition of the antioxidant responsive element (ARE) on target genes, encoding a set of phase II detoxifying enzymes, including HO-1, NQO-1, and GCL [42]. Consistently, we showed that Tak markedly increased the mRNA expression levels of phase II enzymes via Nrf2, and we confirmed that Tak activated Nrf2 through Akt signaling. We thereby concluded that Tak activated phase II enzymes and enhanced cellular redox status, contributing to the improvement of mitochondrial function via Akt-mediated Nrf2 activation.

Abnormal glutamate homeostasis has been reported to be involved in multiple risk factors that contribute to declined cognitive function, such as utilization of clinical drug scopolamine or a lifestyle of long-term HFD intake [18,19,24,25,43,44,45]. Therefore, an in vitro glutamate-challenged HT22 cell model and in vivo scopolamine- and HFD-challenged mice models were used to thoroughly investigate the protective effects of Tak. We found that 5 μM Tak sufficiently prevented glutamate-induced mitochondrial superoxide overproduction and mitochondrial dysfunction, and it inhibited the activation of apoptosis in HT22 cells. Furthermore, we found that the PI3K/Akt pathway was the major upstream kinase that regulated the effects of Tak on phase II enzyme activation and HT22 cell protection against the glutamate challenge. In scopolamine-treated mice, significant cognitive impairment was observed; while mouse locomotor activity was not affected, three behavior tests, including Y-Maze, novel object recognition, and MWM test, all consistently supported the improvement of learning and memory in mice by Tak supplement, accompanied by significantly increased neuronal Akt signaling and redox status. On the other hand, in HFD-treated mice, Tak had no significant effects on mouse metabolic features, such as body weight gain and hyperlipidemia. Interestingly, Tak intervention significantly activated the Akt pathway, induced phase II enzymatic expression, improved mitochondrial function, and inhibited the activation of apoptosis, consistent with the in vitro observations. These data suggest that Tak could specifically work on neurons to exert protective effects via activating Akt-mediated antioxidative systems, and the underlying mechanism of this hippocampus-specific effect requires further study.

## 5. Conclusions

The present study demonstrated that the newly synthesized chalcone analog Tak induced Akt-mediated activation of the Nrf2-phase II enzyme system to improve cellular redox status and enhance mitochondrial function. This beneficial bioactivity could specifically protect neurons against oxidative stress-associated neuronal cell apoptosis in vivo and in vitro. Overall, these findings suggest that Tak may be an attractive agent for the prevention and treatment of neuronal damage-associated diseases.

## Data Availability

The data presented in this study are available in article.

## Supplementary Materials

The following are available online at https://www.mdpi.com/article/10.3390/antiox10111811/s1, Figure S1: Synthetic scheme and structure of 1-(2,3,4-trimethoxyphenyl)-2-(3,4,5-trimethoxyphenyl)-acrylketone; Figure S2: The effect of glutamate on cell viability. HT22 cells were treated with glutamate at 0, 2, 4, 6, 8 µM for 6 (A) and 12 h (B), cell viability was analyzed by MTT; Figure S3: Tak activates phase II enzymes independent of Erk signaling; Figure S4: The effects of Tak on obese mice; Table S1: Detail primer information.
