# Supplementary material for: Chalcone-Derived Nrf2 Activator Protects Cognitive Function via Maintaining Neuronal Redox Status
Source: Antioxidants (Basel). 2021 Nov 15;10(11):1811. doi: 10.3390/antiox10111811 (PMC8615013; doi:10.3390/antiox10111811)
Supplement: Supplementary file 1 [file antioxidants-10-01811-s001.zip › antioxidants-1446419-Supplementary.pdf]

antioxidants

MDPI

Supplementary Materials: Chalcone-Derived Nrf2 Activator Protects Cognitive Function via Maintaining Neuronal Redox Status

Yuting Cui [1,†], Yue Xiong [1,†], Hua Li [1], Mengqi Zeng [1], Yan Wang [1], Yuan Li [2], Xuan Zou [3,4], Weiqiang Lv [1], Jing Gao [1], Ruijun Cao [5], Lingjie Meng [5], Jiangang Long [1], Jiankang Liu [1,6,*] and Zhihui Feng [3,7,*]

[1] Center for Mitochondrial Biology and Medicine, The Key Laboratory of Biomedical Information Engineering of Ministry of Education, School of Life Science and Technology, Xi'an Jiaotong University, Xi'an 710049, China; yutingcui111@stu.xjtu.edu.cn (Y.C.); xw_xjtu@163.com (Y.X.); huali2014@xjtu.edu.cn (H.L.); zmq2010122024@stu.xjtu.edu.cn (M.Z.); wy13572504396@stu.xjtu.edu.cn (Y.W.); gl824705609@stu.xjtu.edu.cn (W.L.); gaojing005@stu.xjtu.edu.cn (J.G.); jglong@mail.xjtu.edu.cn (J.L.)
[2] Institute of Basic Medical Science, Xi'an Medical University, Xi'an 710021, China; yuanli@xiyi.edu.cn
[3] National and Local Joint Engineering Research Center of Biodiagnosis and Biotherapy, The Second Affiliated Hospital of Xi'an Jiaotong University, Shannxi 710004, China; zuseon@xjtu.edu.cn
[4] Shaanxi Provincial Clinical Research Center for Hepatic and Splenic Diseases, The Second Affiliated Hospital of Xi'an Jiaotong University, Shannxi 710004, China
[5] MOE Key Laboratory for Nonequilibrium Synthesis and Modulation of Condensed Matter, School of Science, Xi'an Jiaotong University, Xi'an 710049, China; rjcao@mail.xjtu.edu.cn (R.C.); menglingjie@mail.xjtu.edu.cn (L.M.)
[6] University of Health and Rehabilitation Sciences, Qingdao 266071, China
[7] Frontier Institute of Science and Technology, Xi'an Jiaotong University, Xi'an 710049, China
* Correspondence: j.liu@mail.xjtu.edu.cn (J.L.); zhfeng@mail.xjtu.edu.cn (Z.F.)
† These authors contributed equally to this work.

Table S1. Detail primer information.

Species	Gene	Gene ID	Primer (5'-3')
**Mouse**	HO-1	15368	Forward: AAGCCGAGAATGCTGAGTTCA
			Reverse: GCCGTGTAGATATGGTACAAGGA
**Mouse**	Nqo-1	18104	Forward: AGGATGGGAGGTACTCGAATC
			Reverse: AGGCGTCCTTCCTTATATGCTA
**Mouse**	Gclc	14629	Forward: CTACCACGCAGTCAAGGACC
			Reverse: CCTCCATTCAGTAACAACTGGAC
**Mouse**	Gclm	14630	Forward: AGGAGCTTCGGGACTGTATCC
			Reverse: GGGACATGGTGCATTCCAAAA
**Mouse**	Catalase	12359	Forward: AGCGACCAGATGAAGCAGTG
			Reverse: TCCGCTCTCTGTCAAAGTGTG
**Mouse**	Sod1	20655	Forward: AACCAGTTGTGTTGTCAGGAC
			Reverse: CCACCATGTTTCTTAGAGTGAGG
**Mouse**	Sod2	20656	Forward: CAGACCTGCCTTACGACTATGG
			Reverse: CTCGGTGGCGTTGAGATTGTT
**Mouse**	β-Actin	11461	Forward: GGCTGTATTCCCCTCCATCG
			Reverse: CCAGTTGGTAACAATGCCATGT

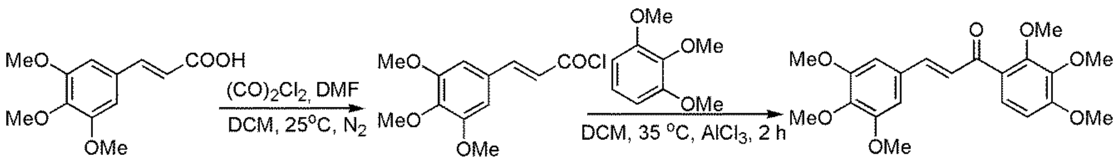

Figure S1. Synthetic scheme and structure of 1-(2,3,4-trimethoxyphenyl)-2-(3,4,5-trimethoxy-phenyl)-acrylketone.

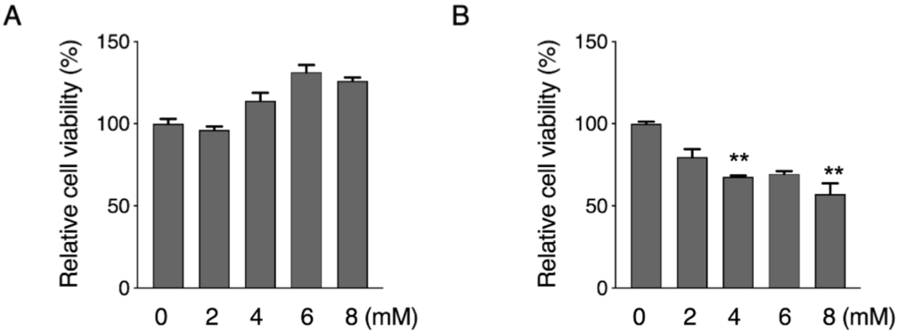

Figure S2. The effect of glutamate on cell viability. HT22 cells were treated with glutamate at 0, 2, 4, 6, 8 μM for 6 (A) and 12 h (B), cell viability was analyzed by MTT.

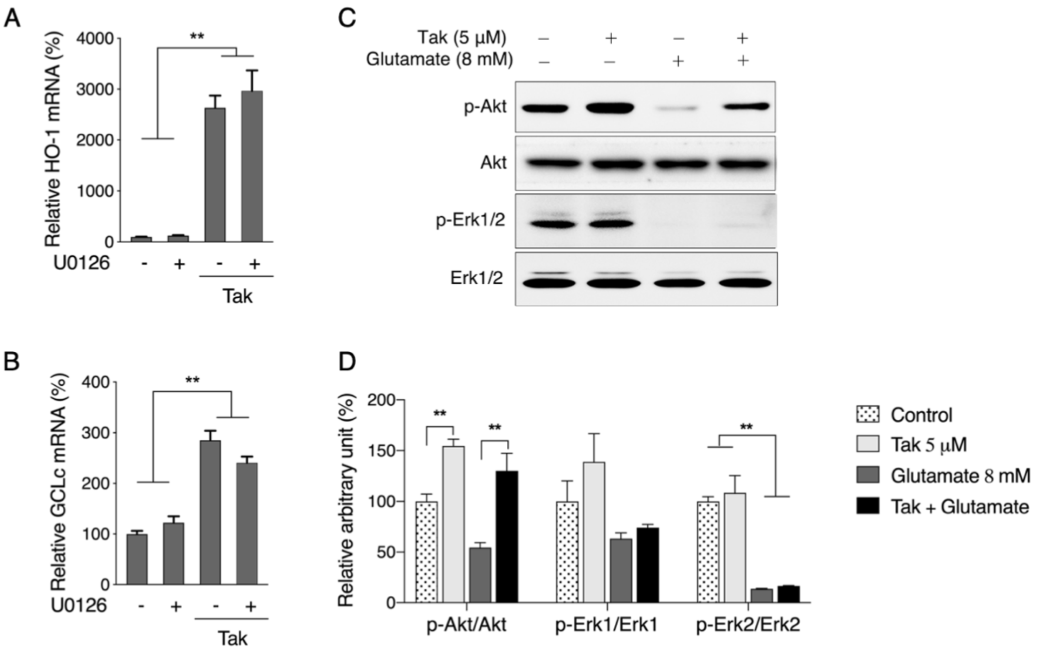

Figure S3. Tak activates phase II enzymes independent of Erk signaling. HT22 cells were treated with Tak for 6 h with or without U0126, and the mRNA expression levels of HO-1 (A) and GCLc (B) were analyzed by qRT-PCR. HT22 cells were pre-treated with 5 μM Tak for 24 h, followed by 8 mM glutamate challenge for 12 h, protein levels of p-Akt and p-Erk1/2 were analyzed by western blot analysis (C: representative images; D: densitometric analysis). The values are presented as the mean ± S.E.M. from at least three independent experiments. *P < 0.05 and **P < 0.01 between the connected groups.

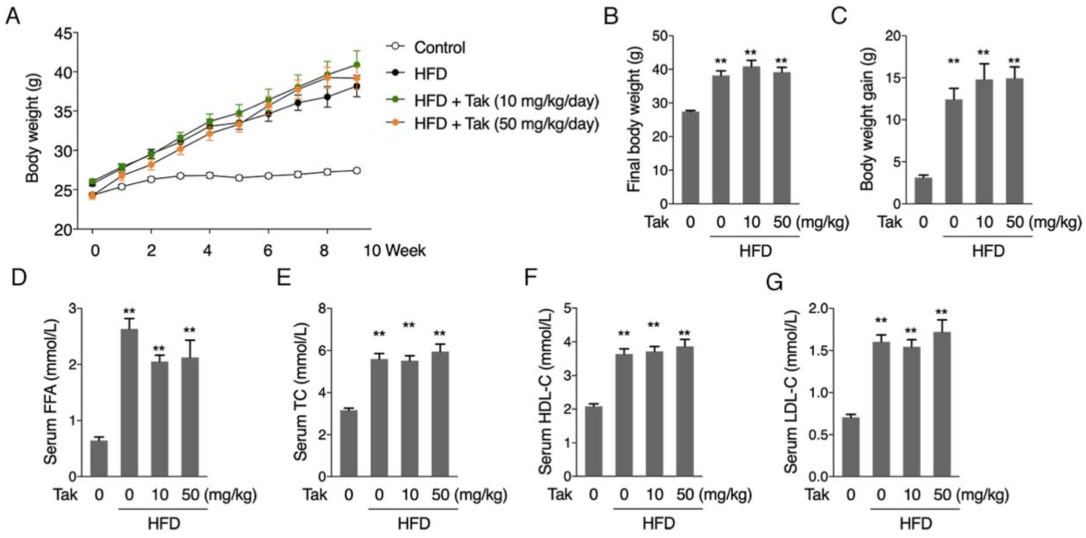

Figure S4. The effects of Tak on obese mice. Mice were fed a chow diet or HFD with or without Tak supplementation at doses of 10 and 50 mg/kg/day for 10 weeks, and the body weight was recorded and serum lipids were analyzed. (A) Body weight curve. (B) Final body weight. (C) Body weight gain. (D) Serum FFA. (E) Serum TC. (F) Serum HDL-c. (G) Serum LDL-c. The values are presented as the mean ± S.E.M., n = 8. *$P < 0.05$ and **$P < 0.01$ between the connected groups.
